# Growth and development in paediatric onset inflammatory bowel disease: Data analysis from the CEDATA registry

**DOI:** 10.1002/jpn3.70544

**Published:** 2026-08-23

**Authors:** Anna Schmidt, Ilse Broekaert, Christoph Hünseler, Jan de Laffolie, CEDATA Study Group

**Affiliations:** ^1^ Department of Paediatrics, Faculty of Medicine and University Hospital Cologne University of Cologne Cologne Germany; ^2^ General Paediatrics and Neonatology, University Children's Hospital, Justus Liebig University Giessen Giessen Germany

**Keywords:** child, Crohn's disease, therapy, ulcerative colitis

## Abstract

**Objective:**

Different therapies may affect the growth and development of children and adolescents with inflammatory bowel disease (IBD) supporting specific therapy algorithms for paediatric Crohn's disease (CD) and ulcerative colitis (UC).

**Methods:**

Growth and development data from the German–Austrian CEDATA‐GPGE registry entered between 2004 and 2021 were analysed for 0–18 year olds with complete follow‐up on diagnoses and therapies. Standard deviation scores (SDS) for height, weight, and body mass index (BMI) were compared between initial diagnosis (t0) and 4‐year follow‐up (t4) within groups using multivariate analysis. Factors included gender, age, disease type, age at diagnosis, and initial therapies.

**Results:**

Data from 450 patients (CD: 303; UC: 147) were analysed. At t0, SDS values for height (mean value [*M*] = −0.37, standard deviation [SD] = 1.07), weight (*M* = −0.57, SD = 0.92), and BMI (*M* = −0.55, SD = 0.95) were below average. The SDS values for weight and BMI increased significantly between t0 and t4 with small effect sizes, whereas the SDS value for height did not. Diagnosis, gender, disease localisation, and remission after 6 months did not influence height SDS. Patients treated exclusively with enteral nutrition (EEN) or azathioprine showed improved weight and BMI development, whereas corticosteroids led to weight gain.

**Conclusion:**

The analysis showed that EEN and disease control significantly improve weight and BMI in children with IBD, SDS‐H catch‐up was only observed in the subgroup with growth impairment at initial diagnosis. Children with severe or recurrent flares, or persistent nutritional deficits, are at increased risk of growth impairment and require close medical monitoring.

## Abstract (highlights)

**What is Known:**

- Growth is a fundamental indicator of the health and well‐being of children and adolescents.
- Children and adolescents with inflammatory bowel disease (IBD) often show impairments in growth and weight gain, which result from a complex interplay of inflammation and restricted nutrient absorption.

**What is New:**

- The choice of treatment and remission status significantly impact growth and weight development in children and adolescents with IBD.
- Early diagnosis and intervention are crucial to avoid long‐term complications, highlighting the importance of effective, personalised therapy.

## Graphical abstract

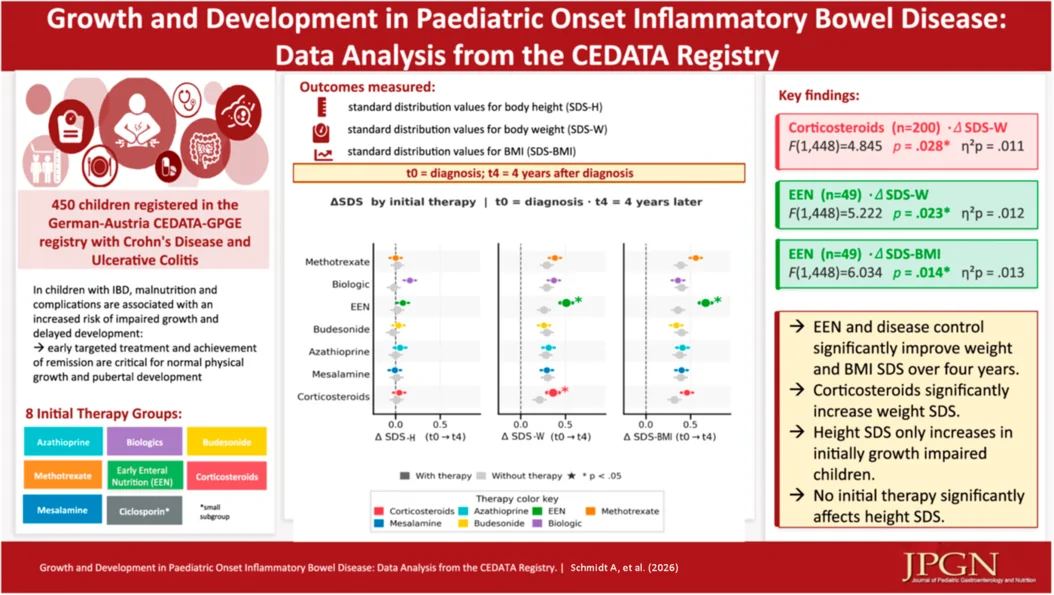

## 1 INTRODUCTION

Inflammatory bowel disease (IBD) is a chronic, relapsing condition affecting the gastrointestinal tract with a peak incidence in early adulthood.^1^ Those who are affected by the disease from an early age suffer longer and have more complications.^2^ The incidence of IBD in children and adolescents has been on the rise in recent years. Intestinal absorption disorders caused by chronic inflammation can lead to significant impairment of linear growth.^3^

Paediatric IBD often manifests during puberty^4^ and the incidence during adolescence is rising.^5^ Growth and developmental disorders are serious complications that are particularly common in Crohn's disease (CD) relative to ulcerative colitis (UC).^6^ The pathogenesis is not fully understood, but immune system malfunction is considered a crucial factor. Environmental factors and modern lifestyle may contribute to the increasing incidence, with familial clustering observed in up to 41% of paediatric cases.^7^

Chronic inflammation often leads to recurring abdominal pain, diarrhoea, and extraintestinal symptoms. Energy deficiency, deficiencies of vitamins and trace elements, and complications such as stenoses or fistulas increase the risk of disorders affecting growth and development. Therefore, early and targeted therapy focusing on remission and ensuring age‐appropriate development is essential.

The primary endpoint of this study is to evaluate different initial therapies (azathioprine, biologics, budesonide, ciclosporin, enteral nutrition [EEN], corticosteroids, mesalamine, and methotrexate) on growth and weight development, whereas secondary outcome measures are influence of gender, disease type, age at diagnosis, as well as disease activity on standard deviation scores for height (SDS‐H), body weight (SDS‐W) and body mass index (BMI) (SDS‐BMI) in children and adolescents with IBD.

## 2 METHODS

### 2.1 Ethics statement

CEDATA‐GPGE® is a pseudonymized multicentre registry with approximately 90 participating clinics in Germany and Austria and is approved by the Ethics Committee of the University Hospital Giessen. Informed consent was given by participants prior to data entry. The research was conducted in accordance with ethical standards outlined in the Declaration of Helsinki.

### 2.2 Data retrieval

Data from paediatric IBD patients registered in the CEDATA‐GPGE registry from January 2004 to June 2021 with a documented course of >6 months and at least two visits from the CEDATA‐GPGE registry were analysed. The CEDATA‐GPGE registry was developed by the IBD working group of the German Society for Paediatric Gastroenterology and Nutrition.^8^

The interval between initial presentation and documentation was at most 14 days. The time points t0 and t4 were defined for the assessment of growth: t0 covers the time from the first visit to the diagnosis and t4 is between 3.75 and 4.25 years after diagnosis. Anthropometric measurements were performed according to standards of the participating centres. Reference data were obtained from growth charts of the Robert Koch Institute.^9^ Standard deviation scores (SDSs) were calculated using the following formula^9^, ^10^: SDS = (*P*‐*μ*)/*σ* (*P*: measured value; *μ*: mean [50th percentile]; *σ*: standard deviation [75th percentile – 25th percentile]/[2 × 0.675]).

Changes in SDS for height (SDS‐H), weight (SDS‐W), and body mass index (SDS‐BMI) between t4 and t0 were analysed as measures of growth over a 4‐year period. Patients received care from multidisciplinary teams, including a dietitian, according to standards of the participating centres.

### 2.3 Statistical analysis

Multivariate analysis was conducted using the quantitative variables SDS‐H, SDS‐W and SDS‐BMI. Factorial variables were gender, disease type, disease course, age at diagnosis, disease activity and initial therapy (biologics, budesonide, azathioprine, methotrexate, corticosteroids, EEN, mesalamine, and ciclosporin).

Analysis of group differences was performed using analysis of variance (ANOVA), after confirming normal distribution with the Shapiro–Wilk test or Q–Q plots and assessing homogeneity of variances using Levene's test. For significant results, additional Bonferroni‐corrected post hoc tests were used to identify specific differences between the groups.

A repeated measures ANOVA was used to examine the change of SDS‐H, SDS‐W and SDS‐BMI over 4 years. A multi‐factorial ANOVA was used to analyse differences in SDS‐H, SDS‐W and SDS‐BMI between t0 and t4 under the influence of disease type and gender. By applying a one‐factor ANOVA, the influence of remission achieved in the first 6 months on the change in body growth over 4 years was investigated.

A multi‐factorial ANOVA was used to analyse the differences in SDS‐H, SDS‐W and SDS‐BMI between t0 and t4 in relation to age at diagnosis and when different therapies are used in the first 6 months after diagnosis. While some groups had sufficiently large case numbers (*n* ≥ 30), other groups contained very small samples (<10). The results for these small groups should be interpreted as exploratory. The significance level was set at 5% (*p* < 0.05). Guidelines for effect strength categories (*η* ^2^ *p*)^11^ are:

- <0.01: negligible effect
- 0.01–0.06: small effect
- 0.06–0.14: moderate effect
- >0.14: large effect.

Statistical analyses were carried out with the programming language R^12^ using the integrated development environment and graphical user interface RStudio.^13^

## 3 RESULTS

Data from 450 IBD patients (303 with CD [67.3%] [125 female {27.8%}], 147 with UC [32.7%] [69 female {15.3%}]) were analysed (Table 1). Figure 1 presents the data processing scheme in a flowchart. Figure 2 shows the changes in SDS‐H, SDS‐W and SDS‐BMI between t0 and t4.

**Table 1:** Information on patient demographics.

		***n***	**Mean age at diagnosis ± SD (y)**	**Min (y)**		**Max (y)**
CD	Male	178	10.9 ± 2.9	2.2		17.2
	Female	125	11.1 ± 2.5	1.0		17.4
UC	Male	78	10.2 ± 3.4	3.3		14.5
	Female	69	10.2 ± 3.0	1.9		15.5
		***n***	**Mean age at remission (y)**	**Min (y)**		**Max (y)**
CD	Male	178	14.4 ± 3.2	2.7		19.5
	Female	125	14.3 ± 2.9	4.7		21.1
UC	Male	78	13.2 ± 3.4	3.7		18.7
	Female	69	12.6 ± 3.5	2.1		17.9
**Extent of the disease**		***n***	**Upper GI tract**	**Small bowel**	**Colon**	**Anorectal**
CD	Male	178	42.1%	60.1%	66.3%	41.6%
	Female	125	37.6%	61.6%	62.4%	49.6%
UC	Male	78	5.1%	5.1%	76.9%	74.4%
	Female	69	7.2%	11.6%	71.0%	68.1%
**Family history of IBD**		***n***	**Yes (%)**	**No (%)**	**N/A (%)**	
CD	Male	178	93 (52.2)	63 (35.4)	21 (11.8)	
	Female	125	66 (52.8)	43 (24.2)	16 (12.8)	
UC	Male	78	44 (56.4)	19 (24.4)	15 (19.2)	
	Female	69	26 (37.7)	22 (31.9)	21 (30.4)	

Abbreviations: CD, Crohn's disease; GI, gastrointestinal; IBD, inflammatory bowel disease; max, maximum observed value; min, minimum observed value; *n*, number; N/A, no information available; SD, standard deviation; UC, ulcerative colitis; *y*, years.

**Figure 1:**
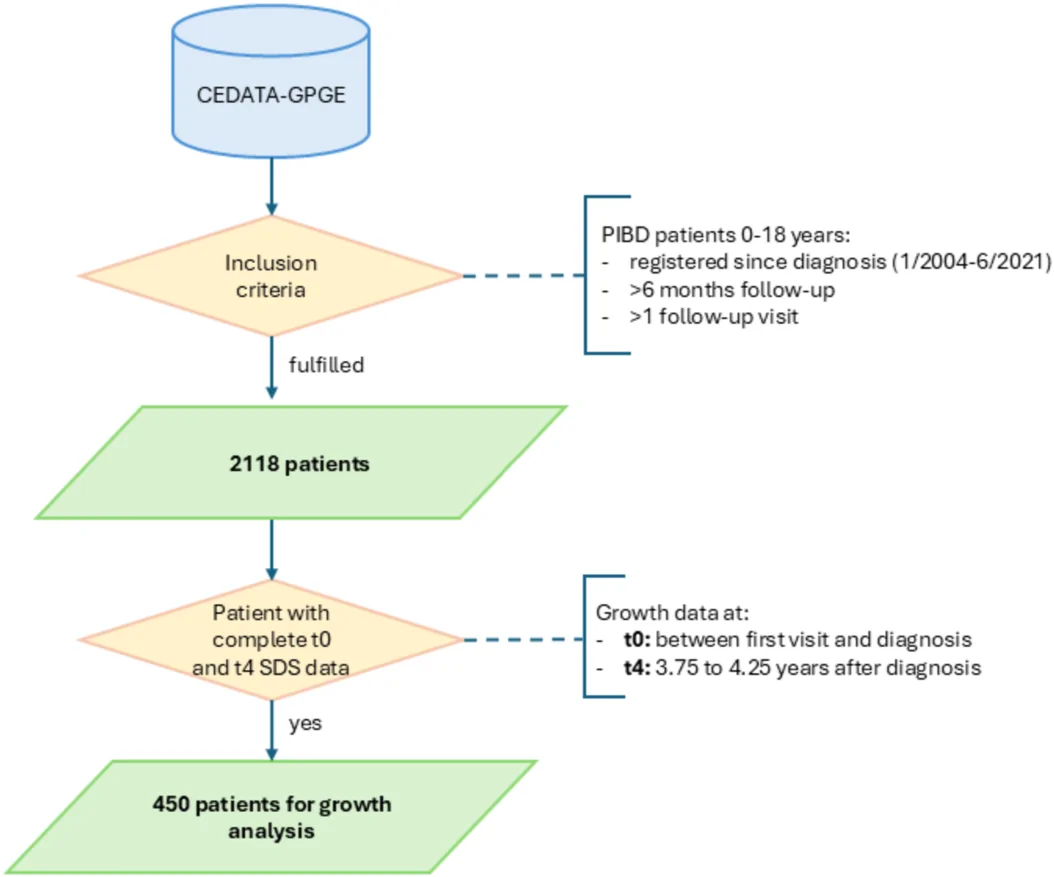
Data processing scheme in a flowchart. CEDATA‐GPGE, a pseudonymized multicentre registry in Germany and Austria; PIBD, pediatric inflammatory bowel disease; SDS, standard deviation scores.

**Figure 2:**
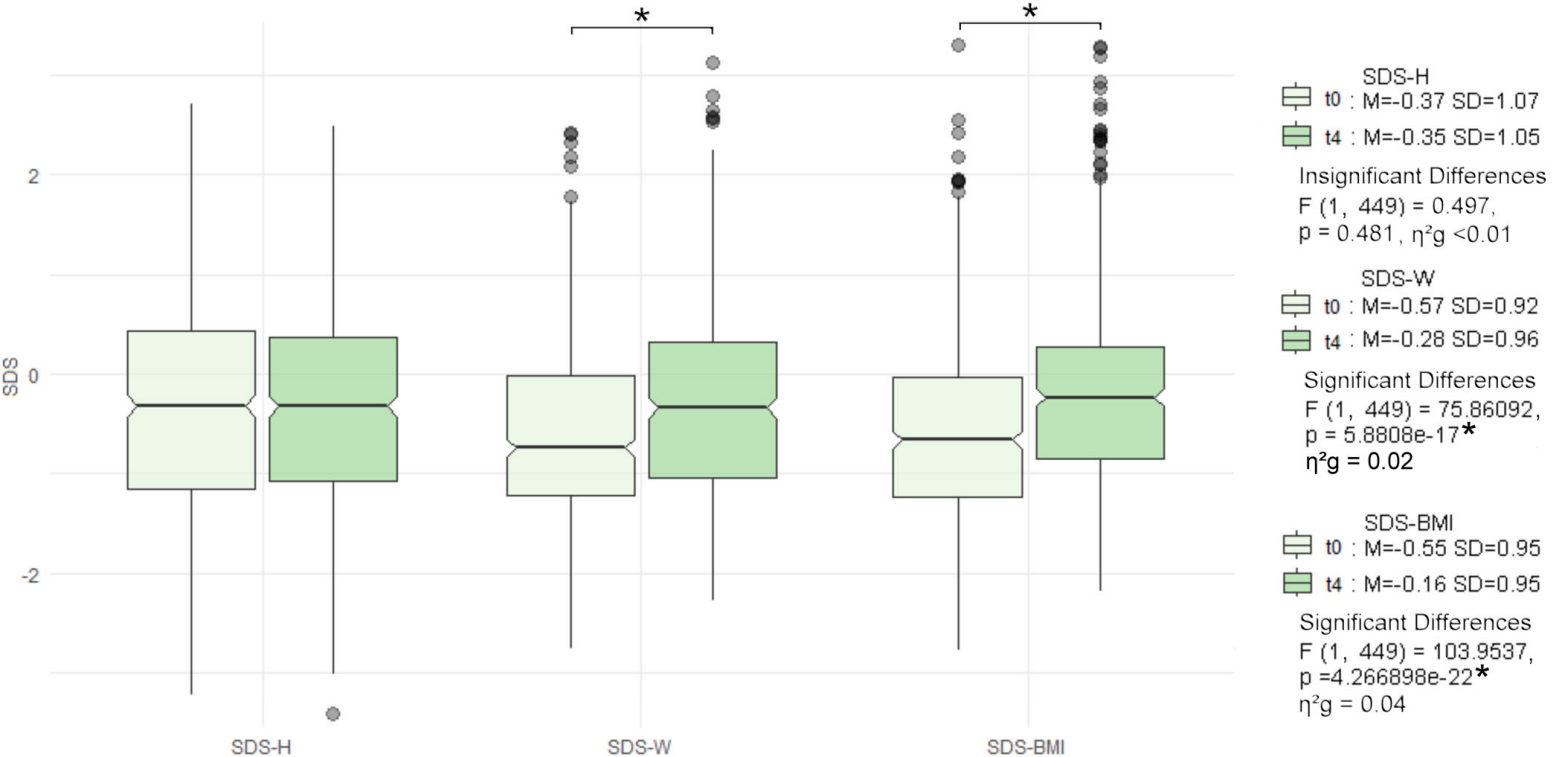
Change in SDS‐height (H), SDS‐weight (W), and SDS‐BMI between t0 and t4 years. BMI, body mass index; SDS, standard deviation scores. *p < 0.05.

To characterise growth impairment more precisely, patients were stratified by SDS‐H at diagnosis using established thresholds. SDS‐H < −2 was present in 33 patients (7.3%), and SDS‐H < −1 in 130 patients (28.9%). Among patients with SDS‐H < −2, mean SDS‐H increased significantly at t4 (*M* = +0.44, SD = 0.51, *p* < 0.001); a similar improvement was observed among those with SDS‐H < −1 (*M* = +0.40, SD = 0.67, *p* < 0.001). No significant change was observed across the cohort as a whole (*M* = +0.02, SD = 0.72), reflecting the fact that most patients did not present with significant growth impairment at diagnosis.

### 3.1 Influence of disease type and gender

Table 2 shows the SDS differences between t0 and t4 according to disease type and gender. Analysis of the SDS‐H difference revealed no significant results with regard to disease type (*F*[1446] = 0.008, *p* = 0.93, *η* ^2^ *p* < 0.01]. Similarly, examination of the influence of gender on the SDS‐H difference showed no significant effects (*F*[1446] = 3.163, *p* = 0.08, *η* ^2^ *p* < 0.01). The SDS‐BMI difference showed no significant differences with regard to disease type (*F*[1446] = 3.301, *p* = 0.07, *η* ^2^ *p* < 0.01] or gender (*F*[1446] = 2.262, *p* = 0.11, *η* ^2^ *p* < 0.01). However, the SDS‐W difference varied significantly by gender (*F*[1446] = 7.196, *p* = 0.01, *η* ^2^ *p* = 0.02), but not by disease type (*F*[1446] = 1.432, *p* = 0.23, *η* ^2^ *p* < 0.01), nor by the interaction of the factors (*F*[1446] = 0.090, *p* = 0.77, *η* ^2^ *p* < 0.01).

**Table 2:** Mean differences in SDS between t0 and t4 stratified for disease entities, gender and initial therapy (*n* > 10).

		*n*	Δ SDS‐H	Δ SDS‐W	Δ SDS‐BMI
Diagnosis	CD	303	*M* = 0.02 SD = 0.73	*M* = 0.31 SD = 0.7	*M* = 0.44 SD = 0.82
	UC	147	*M* = 0.03 SD = 0.72	*M* = 0.24 SD = 0.71	*M* = 0.3 SD = 0.82
	*F*‐value (*p*)		*F*(1448) = 0.033, *p* = 0.855, *η* ^2^ *p* = 0.000074	*F*(1448) = 1.097, *p* = 0.295, *η* ^2^ *p* = 0.0024	*F*(1448) = 2.998, *p* = 0.084, *η* ^2^ *p* = 0.0066
Gender	Male	256	*M* = −0.03 SD = 0.69	*M* = 0.21 SD = 0.65	*M* = 0.34 SD = 0.82
	Female	194	*M* = 0.09 SD = 0.77	*M* = 0.39 SD = 0.76	*M* = 0.46 SD = 0.83
	*F*‐value (*p*)		*F*(1448) = 3.0682, *p* = 0.0806, *η* ^2^ *p* = 0.0068	***F*(1448)** = **6.881, *p*** = 0.00901*, *η* ^2^ *p* = **0.0151**	*F*(1448) = 2.254, *p* = 0.134, *η* ^2^ *p* = 0.005
CD	Male	178	*M* = −0.06 SD = 0.68	*M* = 0.23 SD = 0.67	*M* = 0.4 SD = 0.84
	Female	125	*M* = 0.13 SD = 0.78	*M* = 0.43 SD = 0.74	*M* = 0.5 SD = 0.82
	*F*‐value (*p*)		***F*(1301)** = **4.636, *p*** = 0.0321*, *η* ^2^ *p* = **0.0152**	***F*(1301)** = **5.646, *p*** = 0.0181*, *η* ^2^ *p* = **0.0184**	*F*(1301) = 0.944, *p* = 0.332, *η* ^2^ *p* = 0.0031
UC	Male	78	*M* = 0.03 SD = 0.69	*M* = 0.17 SD = 0.62	*M* = 0.21 SD = 0.78
	Female	69	*M* = 0.03 SD = 0.76	*M* = 0.32 SD = 0.8	*M* = 0.4 SD = 0.85
	*F*‐value (*p*)		*F*(1145) = 0.0004, *p* = 0.983, *η* ^2^ *p* = 0.00000276	*F*(1145) = 1.661, *p* = 0.199, *η* ^2^ *p* = 0.0113	*F*(1145) = 1.967, *p* = 0.163, *η* ^2^ *p* = 0.0134
Corticosteroids	With	200	*M* = 0.01 SD = 0.75	*M* = 0.36 SD = 0.7	*M* = 0.46 SD = 0.8
	Without	250	*M* = 0.04 SD = 0.71	*M* = 0.21 SD = 0.71	*M* = 0.32 SD = 0.84
	*F*‐value (*p*)		*F*(1448) = 0.214, *p* = 0.644, *η* ^2^ *p* = 0.0005	***F*(1448)** = **4.845, *p*** = 0.0282*, *η* ^2^ *p* = **0.0107**	*F*(1448) = 3.42, *p* = 0.065, *η* ^2^ *p* = 0.0076
Mesalamine	With	323	*M* = −0.01 SD = 0.72	*M* = 0.29 SD = 0.73	*M* = 0.4 SD = 0.84
	Without	127	*M* = 0.01 SD = 0.72	*M* = 0.3 SD = 0.66	*M* = 0.39 SD = 0.77
	*F*‐value (*p*)		*F*(1448) = 1.944, *p* = 0.164, *η* ^2^ *p* = 0.0043	*F*(1448) = 0.053, *p* = 0.817, *η* ^2^ *p* = 0.0001	*F*(1448) = 0.001, *p* = 0.974, *η* ^2^ *p* = 0.0000
Azathioprine	With	206	*M* = 0.05 SD = 0.76	*M* = 0.31 SD = 0.75	*M* = 0.41 SD = 0.9
	Without	244	*M* = 0 SD = 0.69	*M* = 0.28 SD = 0.67	*M* = 0.38 SD = 0.75
	*F*‐value (*p*)		*F*(1448) = 0.485, *p* = 0.487, *η* ^2^ *p* = 0.0011	*F*(1448) = 0.186, *p* = 0.666, *η* ^2^ *p* = 0.0004	*F*(1448) = 0.194, *p* = 0.66, *η* ^2^ *p* = 0.0004
Budesonid	With	53	*M* = 0.03 SD = 0.73	*M* = 0.26 SD = 0.79	*M* = 0.34 SD = 1
	Without	397	*M* = −0.03 SD = 0.64	*M* = 0.29 SD = 0.7	*M* = 0.4 SD = 0.8
	*F*‐value (*p*)		*F*(1448) = 0.276, *p* = 0.6, *η* ^2^ *p* = 0.0006	*F*(1448) = 0.115, *p* = 0.735, *η* ^2^ *p* = 0.0003	*F*(1448) = 0.289, *p* = 0.591, *η* ^2^ *p* = 0.0006
EEN	With	49	*M* = 0.08 SD = 0.77	*M* = 0.51 SD = 0.8	*M* = 0.67 SD = 0.8
	Without	401	*M* = 0.02 SD = 0.72	*M* = 0.26 SD = 0.69	*M* = 0.36 SD = 0.82
	*F*‐value (*p*)		*F*(1448) = 0.338, *p* = 0.561, *η* ^2^ *p* = 0.0008	***F*(1448)** = **5.222, *p*** = 0.0228*, *η* ^2^ *p* = **0.0115**	***F*(1448)** = **6.034, *p*** = 0.0144*, *η* ^2^ *p* = **0.0133**
Biologic	With	18	*M* = 0.16 SD = 0.62	*M* = 0.37 SD = 0.74	*M* = 0.36 SD = 0.88
	Without	432	*M* = −0.02 SD = 0.73	*M* = 0.29 SD = 0.71	*M* = 0.4 SD = 0.82
	*F*‐value (*p*)		*F*(1448) = 0.7885, *p* = 0.375, *η* ^2^ *p* = 0.0018	*F*(1448) = 0.217, *p* = 0.642, *η* ^2^ *p* = 0.0005	*F*(1448) = 0.034, *p* = 0.853, *η* ^2^ *p* = 0.0001
Methotrexate	With	16	*M* = 0 SD = 0.79	*M* = 0.38 SD = 0.83	*M* = 0.56 SD = 0.92
	Without	434	*M* = 0.02 SD = 0.72	*M* = 0.29 SD = 0.7	*M* = 0.39 SD = 0.82
	*F*‐value (*p*)		*F*(1448) = 0.013, *p* = 0.909, *η* ^2^ *p* = 0.0000	*F*(1448) = 0.256, *p* = 0.613, *η* ^2^ *p* = 0.0006	*F*(1448) = 0.64, *p* = 0.424, *η* ^2^ *p* = 0.0014

*Note*: Δ: difference in SDS between t0 and t4; *F*(a, b): *F*‐statistic from the ANOVA with *a* (numerator) and *b* (denominator) degrees of freedom; *p*, *p*‐value (significance level); *η* ^2^ *p*: partial eta‐squared, a measure of effect size; *: statistical significance.

Abbreviations: ANOVA, analysis of variance; CD, Crohn's disease; EEN, enteral nutrition; M, mean value; SD, standard deviation; SDS‐BMI, standard deviation scores‐body mass index; SDS‐H, standard deviation scores‐height; SDS‐W, standard deviation scores‐weight; UC, ulcerative colitis.

Bold values denote significant differences (*p* < 0.05).

### 3.2 Disease localisation and age at diagnosis

The change in SDS‐H, SDS‐W and SDS‐BMI between t0 and t4 showed no significant differences in the groups of different disease localisations (upper gastrointestinal [GI] tract, small bowel, colon, and anorectal region) (Table 1). Based on age cut‐offs, a distinction was made between onset <10 years and onset between 10 and 17 years. Two patients with an age at onset >16 years were excluded from the analysis.

No significant differences in SDS‐H (*F*[1446] = 2.692, *p* = 0.10, *η* ^2^ *p* < 0.01) and SDS‐W (*F*[1446] = 2.958, *p* = 0.09, *η* ^2^ *p* < 0.01) were observed between the age groups <10 years and between 10 and 17 years.

The difference in SDS‐BMI (*F*[1446] = 3.909, *p* = 0.049, *η* ^2^ *p* < 0.01) shows statistically significant variations between groups <10 years (*n* = 151, *M* = 0.29, SD = 0.81) and ≥10–<17 years (*n* = 297, *M* = 0.45, SD = 0.83).

### 3.3 Remission after 6 months

The SDS‐H difference of the whole cohort is lower in the group without remission (*M* = −0.05, SD = 0.68, *n* = 197) than in the remission group (*M* = 0.08, SD = 0.75, *n* = 253). The situation is similar for the SDS‐W difference, which is lower in the group without remission (*M* = 0.25, SD = 0.74, *n* = 197) than in the remission group (*M* = 0.32, SD = 0.68, *n* = 253). The SDS‐BMI difference also showed lower values in the group without remission (*M* = 0.38, SD = 0.86, *n* = 197) compared to the remission group (*M* = 0.41, SD = 0.79, *n* = 253). ANOVA confirmed no significant difference in the difference in SDS‐H between time points t0 and t4, *F*[1448] = 3.432, *p* = 0.07, *η* ^2^ *p* < 0.01. Similarly, there is no significant difference in the difference in SDS‐W, *F*(1448) = 1.173, *p* = 0.28, *η* ^2^ *p* < 0.01, and SDS‐BMI, *F*[1448] = 0.162, *p* = 0.69, *η* ^2^ *p* < 0.01 between t0 and t4 in the whole cohort.

### 3.4 Groups with various initial therapies

The differences between the therapies and their combinations with regard to the SDS differences between t0 and t4 were analysed using multi‐factorial ANOVA. Table 2 shows the mean differences in SDS for initial IBD therapies in patient groups *n* ≥ 10 whereas Table S1 shows differences for patient groups <10 and initial combination therapies.

The Bonferroni‐corrected post‐hoc analysis showed a significant difference in the SDS‐H differences only between the groups with (*n* = 8) and without ciclosporin treatment (*p* = 0.02) (Table S1). In the group with ciclosporin therapy, the difference in SDS‐H was lower (*M* = −0.51, SD = 1.17) compared to the group without (*M* = 0.03, SD = 0.71). Analysis of variance homogeneity using Levene's test showed that no equality of variances can be assumed (*p* = 0.02). However, a robust one‐factorial Welch ANOVA confirmed no significant difference between the SDS‐H differences in the groups with and without ciclosporin treatment (*F*[1449] = 1.7335, *p* = 0.23, *η* ^2^ *p* = 0.01). Less growth was observed in boys with CD who received ciclosporin (*n* = 4). Their SDS‐H difference was in the negative range (*M* = −0.92, SD = 0.92) and was lower than in boys with CD without ciclosporin therapy (*M* = −0.04, SD = 0.67). These groups differed significantly (*F*[1176] = 6.735, *p* = 0.01, *η* ^2^ *p* = 0.23) with a moderate effect size. The results for these small groups should be interpreted as exploratory.

Bonferroni‐corrected post‐hoc analysis confirmed a significant difference in SDS‐W differences between the groups with (*n* = 200) and without corticosteroid treatment (*p* = 0.03) (Table 2). The SDS‐W difference was lower in the group without corticosteroid therapy (*M* = 0.21, SD = 0.71) than in the group with (*M* = 0.36, SD = 0.7), although the effect size was small. In addition, the Bonferroni‐corrected post‐hoc analysis showed a significant difference in the SDS‐W differences (difference = 0.352, *p* = 0.02, *η* ^2^ *p* = 0.21, small effect) between EEN (*n* = 40) and corticosteroids (*n* = 191). A significant difference (difference = 0.011, *p* = 0.04, *η* ^2^ *p* = 0.46, small effect) was also found between the treatment combinations “no corticosteroids + EEN + azathioprine” (*n* = 31) and “corticosteroids + no EEN + azathioprine” (*n* = 107). The Bonferroni‐corrected post hoc analysis showed no significant interaction between EEN, azathioprine, and methotrexate with regard to SDS‐W (Table S1).

Boys with UC showed poorer weight gain when treated with azathioprine (*F*[176] = 7.309, *p* = 0.01, *η* ^2^ *p* = 0.088) or biologics (*F*[176] = 8.86, *p* = 0.004, *η* ^2^ *p* = 0.1). The effect size is moderate. The poorer weight gain in boys with UC under azathioprine or biologics therapy influenced their BMI.

The Bonferroni‐corrected post‐hoc analysis confirmed a significant difference in SDS‐BMI differences between the groups with and without EEN (*p* = 0.04) (Table 2). The group with EEN showed a higher SDS‐BMI difference (*M* = 0.67, SD = 0.8) compared to the group without EEN (*M* = 0.36, SD = 0.82), although the effect size was small.

Regarding the action of EEN, azathioprine and methotrexate, the ANOVA test showed a significant difference in SDS‐BMI differences between groups “EEN + azathioprine” (*M* = 0.67, SD = 0.8) and “no EEN + azathioprine” (*M* = 0.36, SD = 0.82) with a positive effect and small effect size (*F*[1442] = 6.027, *p* = 0.01, *η* ^2^ *p* = 0.013). The Bonferroni‐corrected post‐hoc analysis confirmed this difference (*p* = 0.01) and showed a significant interaction between the groups “EEN + azathioprine” (*n* = 38) and “no EEN + azathioprine” (*n* = 168) (difference = 0.415, *p* = 0.03, *η* ^2^ *p* = 0.023).

## 4 DISCUSSION

The aim of this paper is to investigate the growth and developmental trajectories of children and adolescents with IBD, and to identify the risk factors and initial therapies that influence growth development. We show a significant increase in SDS‐W and SDS‐BMI 4 years after diagnosis, while SDS‐H improves only slightly, but not significantly. Children and adolescents continue to grow predominantly at the percentile reached at diagnosis. Disease type and gender had little influence on changes in SDS‐H, SDS‐W or SDS‐BMI. However, there is a significant difference in weight: girls gain more weight than boys, potentially due to specific metabolic or hormonal factors.^14^

Studies by Isa et al.^2^ and El Mouzan et al.^15^ found no influence of gender or IBD type on linear growth. In contrast, Sentongo et al.^16^ showed that males with CD were more likely to have impaired growth. Song et al.,^17^ on the other hand, identified female gender as a risk factor for short stature. Motil et al.^18^ and Sentongo et al.^16^ found that growth failure was more common in CD than in UC. This can be explained by the longer disease duration, the increased difficulty of treatment, and the increased systemic inflammation in CD.^4^ Thus, previous studies show contradictory results regarding the influence of gender and disease type on linear growth, indicating the complexity of the underlying pathophysiological mechanisms.

Our results suggest that achieving remission early may have a positive impact on growth development of children and adolescents with IBD. Patients who achieved remission within 6 months of diagnosis tended to show more favourable growth development. However, our data show that at initial diagnosis, the mean SDS‐H (−0.37 [SD 1.07]) did not show a statistically meaningful reduction compared to the general population, indicating that this specific study population did not exhibit significant growth impairment at initial diagnosis. Significant catch‐up growth was only observed in the subgroup with SDS‐H < −1 at initial diagnosis. While these differences did not reach statistical significance, the trend supports the hypothesis that reducing inflammation early is crucial for optimal physical development. Inflammatory processes affect growth via various mechanisms, such as disrupting intestinal function, increasing the production of proinflammatory mediators, altering gut microbiota and leading to nutritional deficiencies and reduced caloric intake.^7^ In line with previous studies, children in clinical remission were found to have significantly higher SDS‐H than patients with active disease.^19^ These results confirm that reducing inflammatory activity successfully is important not only for controlling the disease itself, but also for ensuring normal growth.

The aims of treatment of IBD in children and adolescents have changed in recent years. In the past, the only objective was to alleviate symptoms, as other options were limited.^20^ Thanks to new therapies for example, the (early) use of biologics and technologies, however, it is now possible to change and significantly improve the natural course of the disease. The most important long‐term treatment goals for patients with IBD are clinical remission, endoscopic healing, restoration of quality of life and the absence of disability. The alleviation of symptoms is defined as an immediate therapeutic goal. The normalisation of serum and faecal biomarkers are considered to be achievable treatment goals in the medium term. In addition, transmural healing in CD and histological healing in UC are becoming important as adjuvant criteria for assessing the intensity of treatment success. Another formal therapeutic goal is the restoration of normal growth.^21^

Corticosteroids are first‐line therapy for moderate to severe UC^22^ and in active luminal CD,^23^ when EEN is not an option. However, they are associated with a wide range of adverse effects, particularly on growth and bone metabolism.^24^ In the present study, corticosteroid use is associated with weight gain, as measured by an increase of SDS‐W over 4 years, presumably due to changes in glucose metabolism. However, anthropometric measurements alone are insufficient for a precise assessment of the effects on nutritional status. Analysing body composition, particularly by measuring fat mass and fat‐free mass, provides a much more reliable indicator.^25^ In this context, Sentongo et al.^16^ observed that cumulative corticosteroid doses correlated positively with obesity development but had no detectable influence on linear growth. In addition, a study by Steiner et al.^26^ shows that high‐dose corticosteroid therapy significantly increases whole‐body protein breakdown and loss in children with CD, even in the short term. This early‐onset catabolic effect could hinder the development of lean body mass in the long term, thereby impacting the physical development of these patients.

Our results show that nutritional therapy, particularly in the form of EEN, has a significant positive effect on weight and BMI development. Girls with CD in particular show an increase in SDS‐W and SDS‐BMI, but not in SDS‐H—they could therefore benefit particularly from nutritional intervention. These effects may be due to improved nutrient absorption by reduced inflammatory activity and increased caloric intake. It is well established that both malnutrition and obesity can impair linear growth.^14^ However, Cameron et al.^27^ reported that in patients newly diagnosed with CD and receiving EEN for induction of remission, a sustained increase in BMI *Z*‐score was observed after 24 months, despite no improvement in height *Z*‐scores. This suggests that weight gain is not necessarily associated with improved linear growth.

In our study, the use of ciclosporin is associated with poorer linear growth development, particularly in male CD patients. The SDS‐H difference is significantly lower in this group, suggesting that severe disease activity may negatively impact growth. The effects should be regarded as exploratory results as the patient group is *n* < 10. As ciclosporin is only used to treat refractory disease, it is an effective therapeutic option for patients with acute UC exacerbations who do not respond to corticosteroid treatment.^28^

The combination of nutritional therapy, azathioprine, and methotrexate led to significant improvements in SDS‐H, SDS‐W, and SDS‐BMI in one patient. Since this observation is based on a single case, it is not statistically valid in the multivariate ANOVA. However, it underscores the need for a larger study to test this combination of therapies.

Only 18 of 450 patients (4.0%) received biologic therapy during the first 6 months after disease onset. However, current clinical practice increasingly favours biologic therapy and reduced corticosteroid which may promote improved growth and development. A limitation of our study is that biologic therapy started beyond the first 6 months after diagnosis was not captured in our analysis; the relationship between initial treatment and catch‐up growth typically occurring within 2 years after diagnosis may be confounded by later biologic initiation, which could itself have contributed to catch‐up growth. Combined with the small number of patients on initial biologic therapy, statistical power to detect a true effect was limited (12%–24% for small‐to‐moderate effect sizes), and the non‐significant finding should be interpreted with caution rather than as evidence of an absence of effect. This underscores the need for effective, personalised therapy guided by prospective clinical studies.

Immunosuppressive therapies can induce clinical and biological remission via mucosal healing in approximately 50% of patients with CD and also have a steroid‐sparing effect.^29^ Patients with EEN‐induced remission showed higher mucosal healing rates and an altered gut microbiota.^30^ Since high levels of proinflammatory cytokines can impair longitudinal growth,^17^ EEN should be considered as a growth‐promoting strategy.

## 5 CONCLUSION

Growth disorders often result from a complex interplay of inflammation and restricted nutrient absorption. We were able to show

- A significant increase in SDS‐W and SDS‐BMI 4 years after diagnosis.
- A positive trend for SDS‐H 4 years after diagnosis; however, growth at initial diagnosis was not significantly impaired.
- That girls gain more weight than boys.
- That the use of corticosteroids is associated with weight gain, as measured by an increase in SDS‐W over 4 years.
- That EEN has a positive effect on weight and BMI development.
- That the SDS‐H difference is significantly lower in the ciclosporin group, suggesting that severe disease activity may negatively affect growth. Unfortunately, the subgroup was far too small.

In order to record the nutritional status more precisely, the CEDATA registry should be amended by adding body composition, as weight and BMI measurement alone are insufficient. Furthermore, it is essential to consider pubertal development in order to correctly assess growth retardation. Nevertheless, assessing malnutrition remains complex, and the influence of pubertal development and skeletal maturation on therapeutic outcomes must be systematically considered. Our data emphasise the importance of achieving early remission highlighting the importance of effective, personalised therapy. Further prospective studies are needed to better understand the impact of disease activity and therapy on growth in IBD and with this understanding to optimise care.

## CONFLICT OF INTEREST STATEMENT

Ilse Broekaert received honorary fees for presentations by Biogen and Pfizer. Jan de Laffolie received honoraria for presentations and lectures by Sanofi, Baxter, Danone, Mirum and Takeda and participated on Advisory Boards not related to IBD by Takeda, Sanofi, Mirum, and Danone. He received funding by Mirum and MetaX. The remaining authors declare no conflicts of interest.

## Supporting information

Supporting File 1
