# Supplementary material for: Growth and development in paediatric onset inflammatory bowel disease: Data analysis from the CEDATA registry
Source: J Pediatr Gastroenterol Nutr. 2026 Aug 23;83(5):810–8. doi: 10.1002/jpn3.70544 (PMC13736134; doi:10.1002/jpn3.70544)
Supplement: Supplementary file 1 [file JPN3-83-810-s001.docx]

| Initial therapy |  | $\boldsymbol{\Delta}$SDS-H | $\boldsymbol{\Delta}$SDS-W | $\boldsymbol{\Delta}$SDS-BMI |
| --- | --- | --- | --- | --- |
| ciclosporin | n=8^#^ | M=-0.51 SD=1.17 | M=-0.03 SD=0.74 | M=0.3 SD=0.98 |
|  | F-value (p) | **F(1,409)=6.385 p=0.012***  **η²p=0.015** | F(1,409)=1.545 p=0.215  η²p<0.01 | F(1,409)=1.545 p=0.215  η²p<0.01 |
| biologics and corticosteroids | n=7^#^ | M=0.03 SD=0.85 | M=0.15 SD=0.89 | M=0.2 SD=0.99 |
|  | F-value (p) | F(1,409)=0.195 p=0.659  η²p<0.01 | F(1,409)=1.582 p=0.209  η²p<0.01 | F(1,409)=0.145 p=0.229  η²p<0.01 |
| biologics and EEN | n=1^##^ | M=0.42 SD=NA | M=0.31 SD=NA | M=0.46 SD=NA |
|  | F-value (p) | F(1,409)=0.001 p=0.974  η²p<0.01 | F(1,409)=0.301 p=0.583  η²p<0.01 | F(1,409)=0.126 p=0.723  η²p<0.01 |
| corticosteroids and EEN | n=9 | M=0.04 SD=0.99 | M=0.3 SD=0.94 | M=0.5 SD=0.87 |
|  | F-value (p) | F(1,409)=0.352 p=0.553  η²p<0.01 | F(1,409)=1.040 p=0.308  η²p<0.01 | F(1,409)=0.419 p=0.518  η²p<0.01 |
| biologics and azathioprine | n=13 | M=0.3 SD=0.57 | M=0.54 SD=0.72 | M=0.49 SD=0.95 |
|  | F-value (p) | F(1,409)=0.502 p=0.479  η²p<0.01 | **F(1,409)=4.894 p=0.02***  **η²p=0.01** | F(1,409)=2.736 p=0.099  η²p<0.01 |
| corticosteroids and azathioprine | n=114 | M=0 SD=0.76 | M=0.19 SD=0.73 | M=0.3 SD=0.88 |
|  | F-value (p) | F(1,409)=0.130 p=0.718  η²p<0.01 | F(1,409)=0.011 p=0.915  η²p<0.01 | F(1,409)=0.002 p=0.962  η²p<0.01 |
| EEN and azathioprine | n=38 | M=0.12 SD=0.76 | M=0.57 SD=0.84 | M=0.75 SD=0.83 |
|  | F-value (p) | F(1,409)=0.645 p=0.422  η²p<0.01 | F(1,409)=3.270 p=0.071  η²p<0.01 | F(1,409)=2.877 p=0.091  η²p<0.01 |
| biologics and methotrexate | n=2^#^ | M=-0.34 SD=1.26 | M=-0.1 SD=0.98 | M=0.09 SD=0.46 |
|  | F-value (p) | F(1,409)=1.450 p=0.229  η²p<0.01 | F(1,409)=0.077 p=0.781  η²p<0.01 | F(1,409)=0.126 p=0.723  η²p<0.01 |
| corticosteroids and methotrexate | n=6^#^ | M=-0.01 SD=1.04 | M=0.04 SD=0.69 | M=0.14 SD=0.64 |
|  | F-value (p) | F(1,409)=0.365 p=0.546  η²p<0.01 | F(1,409)=0.001 p=0.971  η²p<0.01 | F(1,409)=0.183 p=0.669  η²p<0.01 |
| EEN and methotrexate | n=2^#^ | M=-0.27 SD=1.2 | M=0.74 SD=1.32 | M=1.06 SD=0.92 |
|  | F-value (p) | F(1,409)=0.001 p=0.976  η²p<0.01 | F(1,409)=0.015 p=0.904  η²p<0.01 | F(1,409)=0.013 p=0.720  η²p<0.01 |
| azathioprine and methotrexate | n=8^#^ | M=0.09 SD=0.92 | M=0.37 SD=0.97 | M=0.51 SD=1.19 |
|  | F-value (p) | F(1,409)=0.007 p=0.902  η²p<0.01 | F(1,409)=0.002 p=0.966  η²p<0.01 | F(1,409)=0.016 p=0.900  η²p<0.01 |
| biologics and mesalamine | n=7^#^ | M=0.16 SD=0.45 | M=0.48 SD=0.65 | M=0.42 SD=0.81 |
|  | F-value (p) | F(1,409)=0.015 p=0.902  η²p<0.01 | F(1,409)=0.126 p=0.722  η²p<0.01 | F(1,409)=0.160 p=0.689  η²p<0.01 |
| corticosteroids and mesalamine | N=139 | M=-0.06 SD=0.74 | M=0.2 SD=0.73 | M=0.34 SD=0.88 |
|  | F-value (p) | F(1,409)=1.693 p=0.194  η²p<0.01 | F(1,409)=0.402 p=0.526  η²p<0.01 | F(1,409)=0.191 p=0.662  η²p<0.01 |
| EEN and mesalamine | n=37 | M=0.02 SD=0.83 | M=0.43 SD=0.79 | M=059 SD=0.78 |
|  | F-value (p) | F(1,409)=0.186 p=0.667  η²p<0.01 | F(1,409)=3.286 p=0.071  η²p<0.01 | F(1,409)=2.559 p=0.110  η²p<0.01 |
| azathioprine and mesalamine | n=148 | M=-0.01 SD=0.77 | M=0.28 SD=0.75 | M=0.41 SD=0.91 |
|  | F-value (p) | F(1,409)=1.370 p=0.242  η²p<0.01 | F(1,409)=0.020 p=0.886  η²p<0.01 | F(1,409)=0.027 p=0.869  η²p<0.01 |
| methotrexate and mesalamine | n=10^#^ | M=0.06 SD=0.77 | M=0.33 SD=0.66 | M=0.45 SD=0.67 |
|  | F-value (p) | F(1,409)=0.668 p=0.414  η²p<0.01 | F(1,409)=2.329 p=0.128  η²p<0.01 | F(1,409)=1.338 p=0.248  η²p<0.01 |
| mesalamine and ciclosporin | n=3^#^ | M=-0.17 SD=1.43 | M=-0.21 SD=0.63 | M=-0.14 SD=0.42 |
|  | F-value (p) | F(1,409)=0.462 p=0.497  η²p<0.01 | F(1,409)=0.007 p=0.934  η²p<0.01 | F(1,409)=0.038 p=0.845  η²p<0.01 |
| corticosteroids and ciclosporin | n=6^#^ | M=-0.52 SD=1.28 | M=-0.24 SD=0.63 | M=0.09 SD=0.54 |
|  | F-value (p) | F(1,409)=0.078 p=0.781  η²p<0.01 | F(1,409)=2.165 p=0.142  η²p<0.01 | **F(1,409)=5.869 p=0.016***  **η²p<0.01** |
| corticosteroids and EEN and azathioprine | n=7^#^ | M=-0.26 SD=0.9 | M=0.24 SD=1.06 | M=0.6 SD=0.97 |
|  | F-value (p) | **F(1,409)=8.868 p=0.003***  **η²p=0.021** | F(1,409)=0.852 p=0.357  η²p<0.01 | F(1,409)=0.100 p=0.752  η²p<0.01 |
| corticosteroids and azathioprine and methotrexate | n=4^#^ | M=0.23 SD=1.11 | M=0.05 SD=0.58 | M=-0.03 SD=0.46 |
|  | F-value (p) | F(1,409)=0.152 p=0.697  η²p<0.01 | F(1,409)=0.276 p=0.599  η²p<0.01 | F(1,409)=0.420 p=0.517  η²p<0.01 |
| biologics and corticosteroids and mesalamine | n=2^#^ | M=-0.08 SD=0.75 | M=-0.17 SD=0.12 | M=-0.2 SD=0.48 |
|  | F-value (p) | F(1,409)=0.116 p=0.734  η²p<0.01 | F(1,409)=2.376 p=0.124  η²p<0.01 | F(1,409)=1.933 p=0.165  η²p<0.01 |
| corticosteroids and EEN and mesalamine | n=5^#^ | M=-0.28 SD=1.28 | M=0.15 SD=1.28 | M=0.52 SD=1.23 |
|  | F-value (p) | F(1,409)=0.063 p=0.801  η²p<0.01 | F(1,409)=0.269 p=0.604  η²p<0.01 | F(1,409)=0.726 p=0.395  η²p<0.01 |
| biologics and azathioprine and mesalamine | n=5^#^ | M=0.16 SD=0.47 | M=0.45 SD=0.79 | M=0.37 SD=0.98 |
|  | F-value (p) | F(1,409)=0.159 p=0.690  η²p<0.01 | F(1,409)=0.026 p=0.873  η²p<0.01 | F(1,409)=0.092 p=0.761  η²p<0.01 |
| corticosteroids and azathioprine and mesalamine | n=81 | M=-0.07 SD=0.74 | M=0.18 SD=0.74 | M=0.32 SD=0.91 |
|  | F-value (p) | F(1,409)=0.099 p=0.754  η²p<0.01 | F(1,409)=0.063 p=0.802  η²p<0.01 | F(1,409)=0.044 p=0.834  η²p<0.01 |
| EEN and azathioprine and mesalamine | n=28 | M=0.07 SD=0.83 | M=0.53 SD=0.82 | M=0.69 SD=0.81 |
|  | F-value (p) | F(1,409)=0.051 p=0.822  η²p<0.01 | F(1,409)=0.816 p=0.367  η²p<0.01 | F(1,409)=0.686 p=0.408  η²p<0.01 |
| corticosteroids and methotrexate and mesalamine | n=4^#^ | M=0.25 SD=1.11 | M=0.24 SD=0.71 | M=0.24 SD=0.79 |
|  | F-value (p) | F(1,409)=0.418 p=0.518  η²p<0.01 | F(1,409)=2.005 p=0.158  η²p<0.01 | F(1,409)=1.287 p=0.257  η²p<0.01 |
| azathioprine and methotrexate and mesalamine | N=4^#^ | M=0.15 SD=1.12 | M=-0.02 SD=0.59 | M=-0.05 SD=0.46 |
|  | F-value (p) | F(1,409)=0.369 p=0.544  η²p<0.01 | F(1,409)=0.998 p=0.318  η²p<0.01 | F(1,409)=1.931 p=0.165  η²p<0.01 |
| EEN and azathioprine and methotrexate | n=1^##^ | M=0.119 SD=NA | M=0.196 SD=NA | M=0.408 SD=NA |
|  | F-value (p) | F(1,409)= 4.410, p=0.036,  η²p=0.016 | F(1,409)= 5.751,  p=0.017,  η²p=0.013 | F(1,409)= 6.243, p=0.013  η²p=0.015 |
| corticosteroids and EEN and azathioprine and mesalamine | n=4^#^ | M=-0.69 SD=1.02 | M=0.14 SD=1.48 | M=0.68 SD=1.35 |
|  | F-value (p) | F(1,409)=1.698 p=0.193  η²p<0.01 | F(1,409)=0.156 p=0.693  η²p<0.01 | F(1,409)=0.000  p=1.000  η²p<0.01 |

**Supplementary Table 1: Mean differences in SDS between t0 and t4 stratified for initial therapy.** EEN: exclusive enteral nutrition; F(a, b): F-statistic from the ANOVA with a (numerator) and b (denominator) degrees of freedom; M: mean value; n: number; p: p-value; SD: standard deviation; SDS-BMI: standard deviation score for body mass index; SDS-H: standard deviation score for height; SDS-W: standard deviation score for weight; η²p: partial eta-squared, measure of effect size; $\Delta$: difference in SDS between t0 and t4; *: statistical significance; ^#^: These values are only exploratory in nature; ^##^: Statistical significance not interpretable.
